# The Relation between Maternal Work Hours and Primary School Students’ Affect in China: The Role of the Frequency of Mother–Child Communication (FMCC) and Maternal Education

**DOI:** 10.3389/fpsyg.2017.01777

**Published:** 2017-10-12

**Authors:** Huan Zhou, Bo Lv, Xiaolin Guo, Chunhui Liu, Bing Qi, Weiping Hu, Zhaomin Liu, Liang Luo

**Affiliations:** ^1^Collaborative Innovation Center of Assessment toward Basic Education Quality, Beijing Normal University, Beijing, China; ^2^College of Education, Hebei University, Baoding, China; ^3^Key Laboratory of Modern Teaching Technology, Ministry of Education, Shaanxi Normal University, Xi’an, China; ^4^School of Sociology, China University of Political Science and Law, Beijing, China

**Keywords:** primary school students, maternal work hours, children’s affect, mother–child communication, maternal education

## Abstract

**Background:** Although substantial evidence suggests that maternal work hours may have a negative effect on children’s cognitive development, the link between maternal work hours and children’s affect remains unclear. Some studies have observed that non-daytime maternal work hours are associated with more emotional problems among children. However, few studies have focused on the effects of maternal work hours on workdays and non-workdays. Therefore, this study separately investigated the relation between maternal work hours on workdays and on non-workdays and explored the mediating role of the frequency of mother-child communication (FMCC) and the moderating role of maternal education.

**Method:** Using cluster sampling, this study selected 879 students in grades 4–6 at two primary schools in the Hebei and Shandong provinces in China and their mothers as the study subjects. A multi-group structural equation model (SEM) was used to test the relations between maternal work hours, FMCC and children’s affect and the moderating effect of maternal education.

**Results:** (1) Non-college-educated mothers’ work hours on workdays negatively predicted FMCC, but there was no such effect for college-educated mothers; (2) non-workday work hours of all employed mothers negatively predicted FMCC; (3) the FMCC of all employed mothers positively predicted children’s positive affect; (4) the FMCC of college-educated mothers negatively predicted children’s negative affect although there was no such relation for non-college-educated mothers; (5) there was a significant mediating effect of FMCC on the relation between maternal work hours and children’s affect only for non-college-educated mothers; and (6) the workday work hours of non-college-educated mothers positively predicted children’s negative affect, but this correlation was negative for college-educated mothers.

**Conclusion:** Maternal work hours have a marginally significant negative effect on children’s affect through FMCC only for non-college-educated mothers. Compared with non-college-educated mothers, college-educated mothers more easily compensate for the loss of communication opportunities caused by increased work hours on workdays, and children with college-educated mothers benefit more from this communication. However, compensating for the loss of communication opportunities caused by increased work hours on non-workdays is difficult for all employed mothers.

## Introduction

Since the 1960s, the female employment rate has been increasing worldwide, and increasing numbers of women have left family life for work outside the home (Fox et al., 2013). This phenomenon has raised concerns among many researchers and social commentators, who fear that an increase in maternal work hours will have a negative effect on children’s development (Hays, 1996). In most families, the responsibility of caring for children is primarily borne by mothers (Kotila et al., 2013), and mothers spend significantly longer amounts of time with their children than do fathers and other family members (Bianchi et al., 2006; Craig and Mullan, 2011). With the increase in maternal work hours outside the family, women are bound to spend less time and energy accompanying and caring for their children, which can have a negative effect on their children’s development. Thus, in recent years, researchers have sought to uncover the relation between maternal work hours and child development and the underlying mechanisms.

### The Relation between Maternal Work Hours and Child Development

Substantial research evidence suggests that maternal full-time work may have a negative effect on child development. For example, Hill et al. (2005) observed significant negative effects on children’s cognitive outcomes when mothers worked full-time (more than 375 h per quarter) in the 1st year post-birth. Bernal (2008) also observed that during the first 5 years of life, each additional year of full-time work (more than 24 h per week) is associated with a reduction of approximately 1% in children’s cognitive test scores. Although this relation is relatively weak for older children, maternal work hours are nevertheless associated with impaired cognitive development among children (Ruhm, 2008; Lombardi and Coley, 2017). However, these studies also suggest that there are no such negative effects of maternal part-time work on children’s cognitive outcomes. This finding suggests that the number of maternal work hours significantly affects children’s cognitive outcomes.

By contrast, to our knowledge, there is no evidence concerning the direct link between maternal work hours and children’s affect or behavior. However, many studies have demonstrated the significant effects of maternal non-standard work on children’s behavioral and emotional problems. Han et al. (2010) observed that the more often mothers work in the early morning or at night, the more likely their children are to engage in dangerous behaviors; moreover, the younger the children are, the more significant this relation is. In addition, the more years a mother had shift work (particularly shift work during non-daytime hours), the more likely children were to have behavioral problems (Han and Waldfogel, 2007). According to Han and Miller (2009), the more years a mother worked at night, the higher the risk that her children would demonstrate depressive symptoms.

### The Mediating Role of Mother–Child Communication in the Relation between Maternal Work Hours and Child Development

Some researchers suggested that an increase in maternal work hours typically does not have a significant direct effect on child development; rather, the effect occurs because of the reduced time that the mother can spend with her children, particularly the time spent on important parent-child activities (Hsin and Felfe, 2014).

Mother–child communication is the process by which mothers share content, information, opinions, suggestions, emotions, and attitudes with their children in a family to achieve common understanding, trust, and cooperation (Epstein et al., 1993). Such communication is one of the most important methods of achieving appropriate family functioning and is closely related to both maternal work hours and child development (Presser, 2003; Davidson and Cardemil, 2009). Therefore, mother–child communication may be an important mediating variable in the relation between maternal work hours and child development.

Theoretically, maternal time spent with children is restricted by maternal work hours. The parent involvement theory model proposed by Hoover-Dempsey and Sandler (2005) suggested that parents’ perceptions of their available time and energy and parents’ perceptions of demands on their time, particularly those related to employment and other family needs, influence possibilities for involvement in the child’s education. Thus when mothers work longer hours, they perceive having less available time and energy and perceive themselves communicating less frequently with their children (Gettinger and Waters, 1998). Specifically regarding mother–child communication, previous studies have demonstrated that the constraints of maternal work hours are primarily a result of non-standard work (such as working at night, in the early morning, or during vacations). First, non-standard work may reduce the frequency of mother-child communication. Some researchers observed that mothers’ working non-standard hours appears to reduce the opportunity for family dinners (Taht and Mills, 2012) and other interactive activities with their children, such as doing schoolwork, playing games and participating in sports (Han et al., 2010). This situation in turn reduces the chances of mother–child communication. Second, non-standard work may also compromise the quality of mother–child communication. Non-standard work hours are often associated with stressful working conditions, which may make excessive demands on mothers’ energy; consequently, even if mothers can squeeze in time to spend with their children, they will not necessarily have sufficient energy to engage in high-quality mother–child communication (Presser, 2003; Kim et al., 2016). Gassman-Pines (2011) observed that night work or shift work can impair mothers’ physical and mental health and thus their ability to have healthy interactions with their children.

Conversely, parent–child communication is beneficial to child development. First, a parent and child communicating more frequently can promote child development. Direct verbal interaction time between parents and children was determined to benefit the development of children’s linguistic competence, which in turn promotes their cognitive development (Weisleder and Fernald, 2013). In addition, communicating more with parents can promote children’s academic achievements because through communication, parents can help their children develop an objective understanding of their ability and value, convey positive expectations of education, and encourage their children to actively confront difficulties and challenges during the learning process (Davis-Kean, 2005). Moreover, frequent communication is an important means of attaining and strengthening connectedness and intimacy between children and their parents, which is essential for children’s well-being (Kerr et al., 1999). Second, the quality of parent–child communication is also important for the child. Davalos et al. (2005) determined that good parent–child communication enables parents to provide timely feedback to their children on their behavior, thereby improving that behavior and effectively reducing misbehavior and risky behavior. Kim et al. (2016) also suggested that the openness of mother–child communication may negatively predict children’s risky behavior. Furthermore, good parent-child communication was observed to be associated with children’s higher self-esteem and well-being. Similarly, the quantity and quality of parent–child communication were also determined to be important to children’s well-being in China (Wang et al., 2002; Guo et al., 2014).

### The Role of Maternal Education in Moderating the Relations between Maternal Work Hours, Mother–Child Communication and Child Development

Although the results of many studies suggest that an increase in maternal work hours negatively affects child development, not all evidence supports this conclusion. Bernal and Keane (2011) summarized the relevant empirical studies in the United States National Longitudinal Survey of Youth (NLSY), determining that a considerable amount of evidence supports an insignificant or even positive relation between maternal work hours and child development. As a key variable that influences mothers’ time distribution and parenting styles, maternal education may play an important moderating role in this relation.

On the one hand, maternal education can moderate the relation between mothers’ work hours and the frequency of mother-child communication. A study by Hsin and Felfe (2014) demonstrated that the work hours of mothers with a mid-level education (high school, junior college, etc.) may significantly negatively predict the amount of time spent with their children; however, for mothers with either the lowest or highest levels of education, there is no significant relation between the mothers’ education level and the time spent with their children. There are two main underlying reasons for these findings. First, employed women with higher levels of education are more willing to invest time and effort in their children. A report from the United States National Center for Education Statistics reported that college-educated parents are significantly more involved in their children’s educational activities than parents with lower education levels (National Center for Education Statistics [NCES], 2008). A study of 1,053 families also determined that mothers with higher levels of education are more sensitive to the needs of their children, provide them with more positive attention, and invest more time and emotion in them (Huston and Rosenkrantz Aronson, 2005). More recently, a study conducted in China also suggested that mothers with more education are more involved in their children’s education (Wu et al., 2017). Thus, as working hours increase, mothers with higher levels of education may take measures to ensure that they allow sufficient time for their children and have effective interactions with them. By contrast, mothers with relatively low levels of education have low intentions of investing time and energy in their children, and this intention is not influenced by work hours. Second, mothers with higher levels of education are more likely to find jobs with more flexible work hours; that is, they can use their flexible, paid vacation time to be with their children as needed to minimize the negative effects of their work (Galinsky et al., 2011). Therefore, mothers with higher levels of education are more capable of compensating for the loss of time for parent–child activities because of their work. Although mothers with a mid-level education are willing to invest time and energy in their children, their work hours are more restrictive, and it is difficult for them to arrange time to spend time with their children.

However, maternal education can also moderate the relation between the frequency of mother–child communication and child development. Bulgarelli and Molina (2016) observed that the cognitive functioning of children who had been in home-based care increased with the level of maternal education. One important reason is that mothers with higher levels of education engage in more verbal communication with their children and are more adept at using a rich vocabulary during parent–child activities (Bradley and Corwyn, 2002). This contributes to the development of children’s cognitive competence and promotes their understanding of their own and others’ emotions, further promoting the development of their social and emotional skills. Mothers with relatively low levels of education are more likely to adopt a strict and authoritarian mode of parenting (Conger and Donnellan, 2007). They show their children less affection and support and exercise more control and blame during communication (Baumrind, 1994), which is not conducive to healthy child development. Another study conducted in China also suggested that mothers with higher levels of education are more likely to adopt an authoritative parenting style, which may reduce anxiety in children (Gong et al., 2006). In addition, mothers with higher education levels have higher expectations for their children. Because they convey these positive expectations to their children during their interactions, their children are aware that their mothers are paying attention to them and are concerned about them. Consequently, these children are more likely to enjoy interacting with their mothers and benefit more from mother–child activities (Davis-Kean, 2005).

### The Current Study

The results of previous studies indicate that maternal work hours have a significant effect on child development. However, there are some shortcomings in the previous studies regarding the concrete mechanism underlying the relation between maternal work hours and child development.

First, previous studies on the relation between maternal work hours and child development have mostly focused on children’s cognitive development, paying little attention to children’s affect. As an important indicator of children’s psychological adjustment, children’s affect may directly reflect their mental health and subjective well-being, which are key elements of individual development and thus influence important educational outcomes beyond intellectual achievements. The existing evidence indicates that positive affect during childhood can significantly and positively predict children’s future physical and mental health, quality of interpersonal relationships, and professional achievement (Park, 2004). By contrast, depression, anxiety and other types of negative affect can have a significant negative effect on children’s health and well-being, leading to future academic failure, poor social relationships, an increased risk of substance-abuse-related behavior (e.g., smoking, alcoholism) and even suicidal behavior (Otto et al., 2001; Fletcher, 2010). In addition, as primary guardians, mothers play an important role in their children’s affect, which can be significantly influenced by their support, care, and help (Pomerantz et al., 2006; Katz and Hunter, 2007; Eisman et al., 2015). Therefore, it is worthwhile for us to explore the potential effect of increasing maternal work hours on children’s affect.

Second, considerable evidence demonstrates that compared with standard work hours, mothers’ non-standard work hours more negatively affect child development. School-aged children are generally at school during mothers’ regular working hours and typically have more opportunities to communicate with their mothers after school and during holidays. If their mothers work during these times, they miss many opportunities for mother–child communication. However, the non-standard work hours addressed in previous studies are often limited to night or early morning hours (Joshi and Bogen, 2007; Han, 2008; Kim et al., 2016), with few studies examining the relations between maternal work hours on non-workdays and child development (Gassman-Pines, 2011). Thus, to reveal the full picture of the relations between maternal work hours and child development, it is necessary to supplement empirical evidence on non-workdays.

Third, some studies have demonstrated that an increase in maternal work hours does not directly affect child development and that the time that mothers spend with their children, particularly the time spent in important parent–child activities, plays an important role (Hsin and Felfe, 2014). Although extensive evidence has indicated that FMCC is closely related to maternal work hours and child development (Davidson and Cardemil, 2009; Kim et al., 2016), there is nearly no direct evidence that explains the entire picture of these variables. Therefore, clarifying the mediating mechanism of FMCC can help us better understand the relation between maternal work hours and child development.

Fourth, in China, the phenomenon of mothers working outside the home is even more common than in other countries. According to the China Labor Market Development Report 2016 (Lai et al., 2017), the labor participation rate of Chinese women was 64% in 2014, which was significantly higher than the average participation rate of the world’s female labor force overall (50.3%). However, hardly any research has directly explored the potential effect of increasing maternal work hours on child development in the context of Chinese culture. In addition, whereas the culture of Europe and the United States is independence-oriented, the culture of China is interdependence-oriented, and individuals’ sense of worth depends more on others’ judgments of their fulfillment of societal expectations (Kim and Cohen, 2010). Therefore, Chinese mothers’ sense of worth is largely dependent on their children’s accomplishments in school, which is an essential sign of successful parenting in China. To minimize children’s failure and maximize their success in meeting societal expectations, Chinese mothers may be more controlling (over their children’s thoughts, feelings, and behaviors) than mothers in Western countries when communicating with their children (Ng et al., 2014). In Western countries, parental control was observed to be harmful to children’s psychological well-being (Grolnick and Pomerantz, 2009), but it is not necessarily harmful (and can sometimes even be positive) for Chinese children’s psychological well-being (Shek, 2007). Thus, it is worth exploring the relations between mothers’ work hours, FMCC, and children’s affect in the context of China.

To address the limitations of previous studies, we first selected children’s positive and negative affect as the main dependent variables for the study and discussed their relations with maternal work hours. Second, we distinguished the effects of maternal work hours on non-workdays and regular work hours on workdays to explore their respective relations to children’s affect. Third, we examined the potential mediating mechanism of FMCC to test the indirect effect of maternal work hours on children’s affect. Finally, we selected Chinese families as the study subjects to supplement evidence in the context of Chinese culture.

**FIGURE 1:**
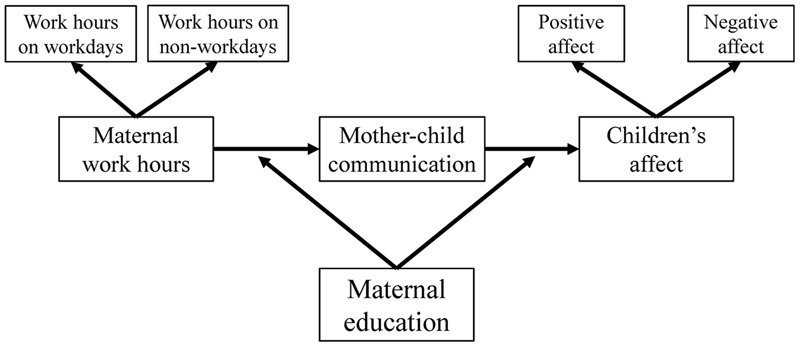
Assumption model of this study.

Previous research has determined that maternal education can moderate the relation between maternal work hours and FMCC and the relation between FMCC and children’s affect. Thus, this study investigated how maternal education moderates the relations between maternal work hours, FMCC, and children’s positive/negative affect. The assumption model of this study is shown in **Figure 1**.

### Hypotheses

First, both theoretical and empirical evidence suggest that maternal work hours restrict mothers’ available time for their children (Hoover-Dempsey and Sandler, 2005; Han et al., 2010; Hsin and Felfe, 2014); thus, our first hypothesis is as follows:

Hypothesis 1: Mothers who work more hours engage in mother–child communication less frequently.

Second, mother–child communication was determined to benefit children’s psychological well-being (Jackson et al., 1998; Kerr et al., 1999); thus, our second hypothesis is as follows:

Hypothesis 2.1: More frequent mother-child communication leads to better children’s affect.

However, Chinese mothers may be more controlling when communicating with their children (Ng et al., 2014), which was determined to be harmful to children’s psychological well-being in Western countries (Grolnick and Pomerantz, 2009). If this is also true in the context of China, the beneficial effect of FMCC may be compromised. This dilemma leads to two competing hypotheses:

Hypothesis 2.2: There is no significant relation between FMCC and children’s affect.
Hypothesis 2.3: More frequent mother-child communication leads to worse children’s affect.

Third, evidence from both Western countries and China suggested that mothers with higher levels of education are willing to spend more time with children (National Center for Education Statistics [NCES], 2008; Wu et al., 2017). Thus, work hours may be less of a constraint for them. Our third hypothesis is as follows:

Hypothesis 3: The negative relation between maternal work hours and FMCC for college-educated mothers is significantly weaker than for non-college-educated mothers.

Finally, evidence from both Western countries and China suggested that mothers with higher levels of education are more likely to adopt a parenting style that is beneficial to children’s psychological well-being (Gong et al., 2006; Conger and Donnellan, 2007). Thus, our fourth hypothesis is as follows:

Hypothesis 4: Children’s affect benefits more from the FMCC of college-educated mothers than of non-college-educated mothers.

## Materials and Methods

### Ethics Statement

All procedures in this study were approved by the Institutional Review Board of the Collaborative Innovation Center of Assessment toward Basic Education Quality, Beijing Normal University. Before the research was conducted, an initial information letter and consent forms were sent to sample schools and then distributed to parents by school staff. The surveys were administered to students after written informed parental consent was received.

### Participants

Cluster sampling was used to select one school from Hebei Province and one school from Shandong Province in China. The education levels of the elementary schools in these two provinces were equivalent to national average levels; for example, the pupil-teacher ratio in Hebei Province in 2014 was 17.84, and in Shandong Province, the ratio was 16.67; the national average was 16.78 (China Statistical Yearbook 2015; Shandong Province Statistical Yearbook, 2015; Hebei Province Statistical Yearbook, 2015). A total of 1,473 students in grades 4–6 from the two primary schools and their mothers were the subjects of this study. In this study, only families with working mothers were selected for two primary reasons. The absence of a working status does not indicate that the mother has abandoned the possibility of working; she may still invest time in work-related activities (such as looking for work or assisting her husband or other relatives with their businesses). These mothers’ “work hours” are not necessarily zero and are difficult to calculate. More importantly, many of the factors related to maternal unemployment are closely linked to child development; for example, mothers’ physical/mental health and alcohol/drug abuse have a significant negative effect on various aspects of child development (Ermisch et al., 2004). Thus, these factors could greatly influence the emotional well-being of children from families with unemployed mothers, which would confuse the research findings.

In this study, 1,473 child questionnaires were distributed. After we excluded questionnaires with a high rate of missing data (i.e., more than one-third of all questions unanswered), 1,350 valid questionnaires remained. Among the families with valid child questionnaires, we eliminated those with missing mothers’ questionnaires or mothers’ questionnaires that were not completed by the mother (*N* = 211), those with incomplete mothers’ questionnaires (i.e., those with more than one-third of all questions unanswered or with missing information regarding work hours or education levels, *N* = 92), and those with unemployed mothers (*N* = 168). Valid data from 879 families were retained (879 child questionnaires and 879 mother’s questionnaires). Among these questionnaires, 475 were from boys (54%), and 404 were from girls (46%); 332 (37.8%) were from fourth graders, 281 (32.0%) were from fifth graders, and 266 (30.3%) were from sixth graders. The children who participated in the study were between the ages of 8 and 14 years, with an average age of 10.20 years (*SD* = 0.93). The mothers were between the ages of 26 and 52 years, with an average age of 36.42 (*SD* = 3.74).

### Measures

#### The Frequency of Mother–Child Communication

*Parental involvement in primary school children education questionnaire (parent version)* (PIPSCEQ) (Wu et al., 2013) is a 29-item parent-reported inventory that was formulated based on Chinese culture. The items described the frequency of parents’ involvement in their children’s educational activities. The inventory contains five subscales representing five important and well-recognized dimensions of involvement in China: (a) parent-school contact, (b) parent–child communication, (c) learning assistance, (d) parent–child activity, and (e) home monitoring. This study used the six items for the parent–child communication dimension from the PIPSCEQ to measure the frequency of mother–child communication. These items describe representative content of parent–child communication. Participants were required to rate the frequency of the condition that each item described on a 4-point Likert-type scale ranging from never (1) to always (4). An example of the items is “When my child comes to tell me about his/her interests, I stop what I am doing to listen to him/her.” The questionnaire was to be taken home by the students after school and completed by their mothers.

The reliability and validity of the questionnaire were tested using the data in this study. The internal consistency coefficient (Cronbach’s *α*) of the questionnaire was 0.723. The results of a confirmatory factor analysis showed that the fit indexes of the model were acceptable: *χ2* = 39.245, *df* = 9, *p* < 0.001, *χ2/df* = 4.361, *TLI* = 0.947, *CFI* = 0.968, *RMSEA* = 0.057. Therefore, the parent-child communication questionnaire had good structural validity and reliability in this study (Marsh et al., 2004). As a latent variable measured by the six themes, FMCC was included in the structural equation model (SEM) for statistical analysis.

#### Children’s Affect

The Positive and Negative Affect Scale (PANAS) compiled by Watson et al. (1988) was used to measure children’s positive/negative affect. The scale includes nine adjectives describing positive affect and nine adjectives describing negative affect (e.g., happy, angry). Subjects were requested to respond with an estimate of the extent to which they had experienced the emotions described by these words in the most recent week based on their own feelings. They responded on a 5-point Likert-type scale, with 1 representing “very slight or not at all” and 5 representing “very strong.” The underlying construct of the self-reported PANAS has been shown to be stable from childhood to adulthood (Allan et al., 2015), and the scale has been validated for Chinese primary students (e.g., Lv et al., 2016).

The reliability and validity of the scale in this study were tested using the study data. The internal consistency coefficients (Cronbach’s *α*) of the positive affect scale and the negative affect scale were 0.841 and 0.797, respectively. The results of the confirmatory factor analysis indicated that the fit indexes of the model were acceptable after the residuals of four pairs of items were allowed to be correlated: *χ2* = 434.792, *df* = 130, *p* < 0.001, *χ2/df* = 3.345, *TLI* = 0.936, *CFI* = 0.946 and *RMSEA* = 0.047. Therefore, the PANAS had good structural validity and reliability in this study (Marsh et al., 2004). As latent variables measured by the nine statements of the relative dimensions, children’s positive/negative affect was included in the SEM for statistical analysis.

#### Maternal Work Hours

On the mother’s questionnaire, two questions were used to examine maternal work hours: one question about work hours on workdays and another about work hours on non-workdays. (1) The question that examined work hours on workdays was “Your average work hours per standard workday in the past year were: 1 = unemployed, 2 = 0–2 h, 3 = 3–5 h, 4 = 6–8 h, 5 = 9–10 h, and 6 = 11 h or more.” (2) The question that examined overtime work hours on non-workdays was “The number of days that you worked overtime on Saturdays, Sundays, and national holidays in the past year was: 1 = 0 days, 2 = 1–30 days, 3 = 31–60 days, 4 = 61–90 days, and 5 = 91 days or more.” Based on the answer to the first question, unemployed mothers (mothers who chose “1” [*N* = 168]) were excluded from the study.

### Maternal Education

Maternal education level was obtained from the mother’s questionnaire. The survey question was as follows: “Your education level is 1 = primary or below, 2 = middle school, 3 = high school/vocational high school, 4 = junior college/vocational school, 5 = undergraduate or 6 = master’s level or above.”

#### Demographic Variables

The children’s gender and grade were obtained from the child’s questionnaire. Household income was obtained from the mother’s questionnaire; the question was as follows: “The annual income of your family last year (annual income is the sum of the income of all members of the family, including the actual after-tax amount of wages, bonuses, and subsidies and the converted RMB amount of physical objects of value [such as food] that the family receives): 1 = 3,600 yuan or below, 2 = 3,601–7,200 yuan, 3 = 7,201–14,000 yuan, 4 = 14,001–30,000 yuan, 5 = 30,001–50,000 yuan, 6 = 50,001–100,000 yuan, 7 = 100,001–200,000 yuan, 8 = 200,001–300,000 yuan, and 9 = 300,001 yuan or above.” The number of children was obtained from the mother’s questionnaire; the question was as follows: “At present, how many children do you have? 1 = one child, 2 = two children, 3 = three children, 4 = four children or more.”

### Procedure

A group test was used to conduct this survey. We took the natural class as a group, and the average number of students in each class (group) was 72. The students completed the questionnaires under the guidance of the researchers (2 researchers for each class) in their classes. The introduction to this survey was presented at the beginning of the questionnaire, and one researcher in each class loudly read the introduction to ensure that the students understood how to answer the questions. The survey lasted 20 min, and the questionnaires were collected on site.

After the questionnaires were collected, the researchers distributed the mother’s questionnaires to the children in the classroom. The children were requested to take the mother’s questionnaires and a letter describing the purpose and process of the study home to their mothers, have their mothers complete the questionnaire, bring the completed questionnaires back to school, and hand them in to the researchers the next day.

### Data Analysis

SPSS 22.0 and Mplus 7.4 were used to manage and analyze the data. A multi-group SEM of the latent variables was used to verify the validity of the model assumptions. The missing values among the responses of our valid subjects in each variable did not exceed 1.5%, and Little’s MCAR test showed that the missing data for all variables were randomly distributed (*ps* > 0.05). The missing values were processed using full information maximum likelihood estimation (FIML) (Little and Rubin, 1987).

## Results

### Full-Sample Model

We first estimated a baseline relation model between maternal work hours, FMCC and children’s affect for the full sample after controlling for maternal education, household income, children’s gender and grade, and the number of children in the family. The fit indexes of the model were acceptable and are as follows: *χ2* = 733.907, *df* = 379, *χ2/df* = 1.936, *TLI* = 0.930, *CFI* = 0.937 and *RMSEA* = 0.033.

The means and standard deviations of the major variables of all subjects and their Pearson correlation coefficients are shown in **Table 1**. FMCC exhibited significant negative correlations with mothers’ work hours on workdays (*r* = -0.151, *p* < 0.001) and non-workdays (*r* = -0.188, *p* < 0.001). In addition, FMCC was positively correlated with children’s positive affect (*r* = 0.110, *p* < 0.01) and negatively correlated with children’s negative affect (*r* = -0.071, *p* < 0.05). However, maternal work hours on both workdays and non-workdays had no significant direct relation to children’s positive or negative affect.

**Table 1:** The means and standard deviations of the major variables and the correlation coefficients among them (*N* = 879).

	1	2	3	4	5	6	7	8	9	10
(1) FMCC	1									
(2) Maternal work hours on workdays	-0.151***	1								
(3) Maternal work hours on non-workdays	-0.188***	0.354***	1							
(4) Children’s positive affect	0.110**	-0.026	-0.029	1						
(5) Children’s negative affect	-0.071*	0.043	0.019	-0.145***	1					
(6) Children’s gender	-0.001	-0.021	-0.041	-0.045	-0.065	1				
(7) Children’s grade	-0.075*	0.047	0.073*	-0.160***	0.043	0.001	1			
(8) Maternal education	0.173***	-0.242***	-0.210***	0.079*	-0.098**	-0.001	-0.093**	1		
(9) Household income	0.109**	-0.054	0.034	0.019	-0.093**	-0.029	-0.060	0.244***	1	
(10) Number of children	-0.112**	0.054	-0.025	-0.081*	0.032	0.193**	0.053	-0.279**	-0.066	1
*M*	3.360	4.350	2.520	3.804	2.085	0.460	4.920	3.180	4.340	1.471
*SD*	0.426	0.721	1.362	0.742	0.671	0.499	0.822	1.184	1.673	0.543

*^∗^*p* < 0.05; ^∗∗^*p* < 0.01; ^∗∗∗^*p* < 0.001.*

### Moderation by Maternal Education: Multi-Group Analysis

All subjects were divided into the following two groups according to the level of maternal education: a group of college-educated mothers (maternal education level of junior college or above, *N* = 323, 36.75%) and a group of non-college-educated mothers (maternal education level below junior college, *N* = 556, 63.25%). The reliability and structural validity were good for these two groups. (1) For PIPSCEQ (parent–child dimension), the Cronbach’s *α* was 0.720 (college-educated mothers) and 0.731 (non-college-educated mothers). Fit indexes of the CFA model were also acceptable: *χ2* = 25.422, *df* = 9, *p* < 0.001, *χ2/df* = 2.825, *TLI* = 0.948, *CFI* = 0.969 and *RMSEA* = 0.057 (college-educated mothers); *χ2* = 25.422, *df* = 9, *p* < 0.001, *χ2/df* = 2.825, *TLI* = 0.948, *CFI* = 0.969 and *RMSEA* = 0.057 (non-college-educated mothers). (2) For PANAS, the Cronbach’s *α* of the positive affect scale was 0.846 (college-educated mothers) and 0.838 (non-college-educated mothers), and the Cronbach’s *α* of the negative affect scale was 0.800 (college-educated mothers) and 0.783 (non-college-educated mothers). Fit indexes of the CFA model were also acceptable: *χ2* = 231.877, *df* = 130, *p* < 0.001, *χ2/df* = 1.784, *TLI* = 0.934, *CFI* = 0.944 and *RMSEA* = 0.049 (college-educated mothers); *χ2* = 316.804, *df* = 130, *p* < 0.001, *χ2/df* = 2.437, *TLI* = 0.923, *CFI* = 0.935 and *RMSEA* = 0.051 (non-college-educated mothers).

Controlling for household income, children’s gender, grade, and the number of children, the study used multi-group SEM to examine the relations between maternal work hours, FMCC and children’s positive/negative affect in these two groups of mothers. At first, the configural invariance between the two groups was tested by defining these two groups in same model but defining no parameter as equal. The fit indexes of this model were good and are as follows: *χ2* = 1157.894, *df* = 758, *χ2/df* = 1.528, *TLI* = 0.921, *CFI* = 0.929 and *RMSEA* = 0.035. The results indicated that the structural relations between variables of the two groups were equivalent. Then, by defining the factor loadings of the measurement models of the two groups of subjects as equal, we tested whether the measurement models of the two groups were equivalent. The fit indexes of this model were good and are as follows: *χ2* = 1178.402, *df* = 779, *χ2/df* = 1.513, *TLI* = 0.923, *CFI* = 0.928 and *RMSEA* = 0.034. The test results indicated that the fit indexes of the models did not significantly change, *Δχ2* = 20.508 (*df* = 21, *N* = 879, *p* > 0.05), indicating that the measurement models of the two groups were equivalent.

**FIGURE 2:**
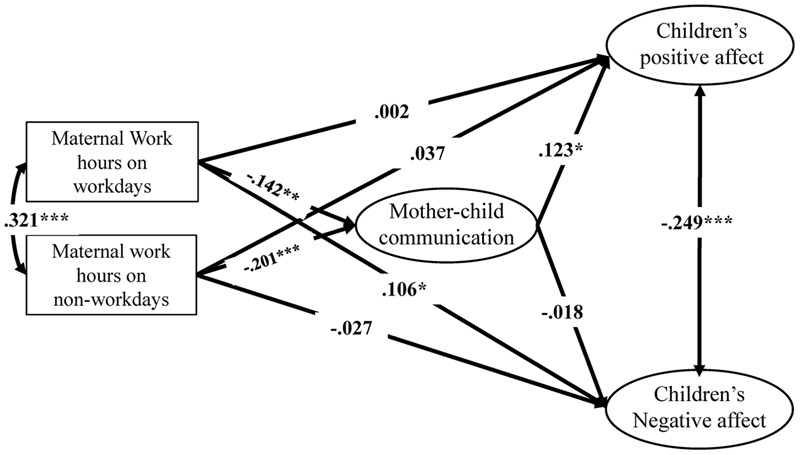
The mediating effect of non-college-educated mothers’ FMCC on the relation between maternal work hours and children’s positive/negative affect.

The mediating model of the non-college-educated mothers is shown in **Figure 2** and **Table 2**. Maternal work hours on both workdays (*β* = -0.142, *p* < 0.01) and non-workdays (*β* = -0.201, *p* < 0.001) significantly and negatively predicted FMCC. FMCC significantly and positively predicted children’s positive affect (*β* = 0.123, *p* < 0.05) but did not significantly predict children’s negative affect (*β* = -0.18, *p* > 0.05). On workdays, the mediating effect of FMCC on the relation between maternal work hours and children’s positive affect was marginally significant (Sobel, 1982): the value of the mediating effect was -0.017 (*Sobel Z* = -1.71, *p* = 0.087). However, the effect was not significant for children’s negative affect: the value of the mediating effect was 0.003 (*Sobel Z* = 0.301, *p* > 0.05). On non-workdays, the mediating effect of FMCC on the relation between maternal work hours and children’s positive affect was marginally significant: the value of the mediating effect was -0.025 (*Sobel Z* = -1.890, *p* = 0.059). However, it was not significant for children’s negative affect: the value of the mediating effect was 0.004 (*Sobel Z* = 0.302, *p* > 0.05).

**Table 2:** Standardized direct, indirect, and total effects of maternal work hours on children’s positive/negative affect among non-college-educated mothers (*N* = 556).

	FMCC	Children’s positive affect			Children’s negative affect		
	Direct effect	Direct effect	Indirect effect	Total effect	Direct effect	Indirect effect	Total effect
Independent variables							
Maternal work hours on workdays	-0.142**	0.002	-0.017	-0.015	0.106*	0.003	0.109*
Maternal work hours on non-workdays	-0.201***	0.037	-0.025	0.012	-0.027	0.004	-0.023
**Mediating variables**							
FMCC	–	0.123*	–	–	-0.018	–	–
**Control variables**							
Children’s gender	0.001	-0.074	–	–	-0.057	–	–
Children’s grade	-0.052	-0.110*	–	–	-0.054	–	–
Household income	0.113*	0.001	–	–	-0.097*	–	–
Number of children	-0.113*	-0.074	–	–	-0.003	–	–

*^∗^*p* < 0.05; ^∗∗^*p* < 0.01; ^∗∗∗^*p* < 0.001. FMCC, the frequency of mother–child communication.*

**FIGURE 3:**
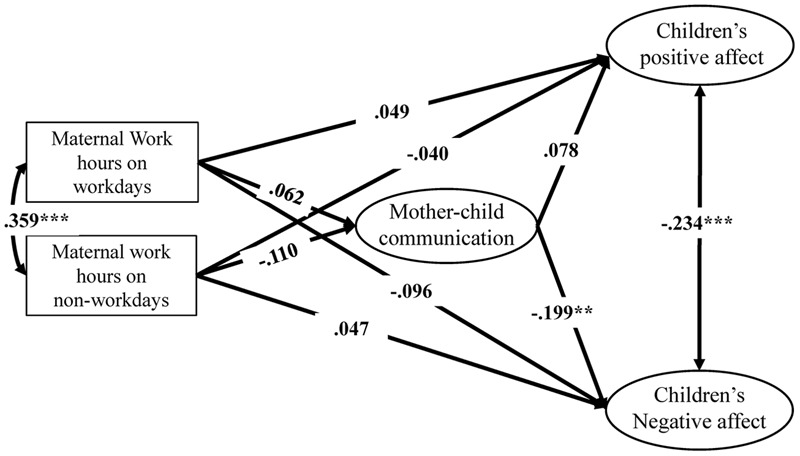
The mediating effect of college-educated mothers’ FMCC on the relation between maternal work hours and children’s positive/negative affect.

The mediating model of college-educated mothers is shown in **Figure 3** and **Table 3**. Neither maternal work hours on workdays (*β* = 0.062, *p* > 0.05) nor maternal work hours on non-workdays (*β* = -0.110, *p* > 0.05) exhibited a significant relation to FMCC. FMCC significantly and negatively predicted children’s negative affect (*β* = -0.199, *p* < 0.01) but did not significantly predict children’s positive affect (*β* = 0.078, *p* > 0.05). On workdays, the mediating effect of FMCC on the relation between maternal work hours on workdays and children’s positive/negative affect was not significant; the values of the mediating effect were 0.005 (*Sobel Z* = 0.689, *p* > 0.05) and -0.012 (*Sobel Z* = -0.834, *p* > 0.05), respectively. Similarly, the mediating effect was also not significant on non-workdays: the values of the mediating effects were -0.009 (*Sobel Z* = -0.907, *p* > 0.05) and 0.022 (*Sobel Z* = 1.354, *p* > 0.05), respectively.

**Table 3:** Standardized direct, indirect, and total effects of maternal work hours on children’s positive/negative affect among college-educated mothers (*N* = 323).

	FMCC	Children’s positive affect			Children’s negative affect		
	Direct effect	Direct effect	Indirect effect	Total effect	Direct effect	Indirect effect	Total effect
**Independent variables**							
Maternal work hours on workdays	0.062	0.049	0.005	0.054	-0.096	-0.012	-0.108
Maternal work hours on non-workdays	-0.110	-0.040	-0.009	-0.049	0.047	0.022	0.069
**Mediating variables**							
FMCC	–	0.080	–	–	-0.199**	–	–
**Control variables**							
Children’s gender	0.044	0.061	–	–	-0.118	–	–
Children’s grade	-0.049	-0.248***	–	–	0.124*	–	–
Household income	0.087	-0.038	–	–	-0.060	–	–
Number of children	-0.076	-0.013			0.030		

*^∗^*p* < 0.05; ^∗∗^*p* < 0.01; ^∗∗∗^*p* < 0.001. FMCC, the frequency of mother–child communication.*

### The Moderating Role of Maternal Education

We tested a series of nested models using multi-group analysis to further determine which paths were different between college-educated mothers and non-college-educated mothers. On each occasion, we constrained one path coefficient of the structural model and compared its model fit with the fully unconstrained model (all path coefficients of the structural model being free to vary across groups). The test results indicated that three path coefficients demonstrated significant differences. (1) The negative effect of maternal workday work hours on FMCC for the non-college-educated mothers (*β* = -0.142, *p* < 0.01) was significantly greater than for the college-educated mothers (*β* = 0.062, *p* > 0.05); *Δχ2* = 5.370 (*df* = 1, *p* < 0.05). This finding was consistent with Hypothesis 3. (2) The negative effect of college-educated mothers’ FMCC on children’s negative affect (*β* = -0.199, *p* < 0.01) was significantly greater than the effect of non-college-educated mothers (*β* = -0.018, *p* > 0.05); *Δχ2* = 4.125 (*df* = 1, *p* < 0.05). This finding was consistent with Hypothesis 4. (3) For the non-college-educated mothers, maternal work hours on workdays significantly directly and positively predicted children’s negative affect (*β* = 0.106, *p* = 0.05) whereas for college-educated mothers, maternal work hours on workdays directly and negatively predicted children’s negative affect (*β* = -0.096, *p* > 0.05); *Δχ2* = 5.632 (*df* = 1, *p* < 0.05).

## Discussion

This study explored the relations between maternal work hours, mother–child communication, and children’s positive/negative affect as well as the moderating role of maternal education in the context of Chinese culture. Generally, the correlation coefficients among variables (**Table 1**) support Hypothesis 1 and Hypothesis 2.1: FMCC exhibited significant negative correlations with maternal work; FMCC was positively correlated with children’s positive affect and negatively correlated with children’s negative affect. The correlation coefficients among the variables were significant but not very high, perhaps because there are some factors that could moderate the relations between the variables, such as the role of fathers (Milkie et al., 2015), use of center are (NICHD Early Child Care Research Network, 2006; Bulgarelli and Molina, 2016), maternal work schedules (Han and Miller, 2009), and maternal education (Davis-Kean, 2005; Hsin and Felfe, 2014; Wu et al., 2017).

Thus, this study selected one of the important moderating factors, maternal education, to explore the delicate relations among maternal work hours, FMCC, and children’s affect separately in two groups of mothers: college-educated mothers and non-college-educated mothers. The results of multi-group SEM analyses indicated that the moderating effect of maternal education was mainly demonstrated in three areas: (1) the relation between maternal work hours on workdays and FMCC; (2) the relation between FMCC and children’s negative affect; and (3) the relation between maternal work hours on workdays and children’s negative affect.

First, maternal work hours on workdays significantly and negatively predicted the FMCC of non-college-educated mothers; no such effect was observed in the group of college-educated mothers. Thus, on workdays, Hypothesis 1 was supported only for non-college-educated mothers, and the difference between college-educated mothers and non-college-educated mothers is consistent with Hypothesis 3. According to the parent-involvement-theory model proposed by Hoover-Dempsey and Sandler (2005), parents’ perceived amounts of available time and energy can significantly affect their involvement with their children and thus influence children’s development. Consequently, when working mothers work longer hours, they perceive themselves to have less available time and energy, which can hinder their interactions with their children and in turn, hinder the children’s developmental outcomes (Gettinger and Waters, 1998). However, our results indicated that this effect vanished among college-educated mothers. There are two possible reasons for this finding. First, highly educated mothers are more likely to find jobs with flexible work hours, and they can better schedule work and rest periods based on their children’s needs (Galinsky et al., 2011). Consequently, when children fall ill, exhibit declining academic performance, or have other emergencies, college-educated mothers are more likely to spend time with them. Second, Walker et al. (2005) noted that the available time and effort perceived by parents are not the only factors that influence childrearing behavior; another factor with a similar influence on childrearing is parents’ perceptions of their knowledge and skills. Thus, although time and energy may limit the time that mothers spend with their children, this limit is lower when mothers perceive that they have a relatively high level of knowledge and skills. These mothers are more likely to believe that their communication with their children is beneficial to their children’s development, and they are more motivated to find ways to find time to communicate with their children. Because mothers with high levels of education have relatively high levels of knowledge and skills, they are more proactive in addressing the conflicts between their work and the need for mother–child communication.

Second, FMCC among college-educated mothers significantly and negatively predicted children’s negative affect; however, no such effect was observed for the non-college-educated mothers. This result partially supported Hypothesis 2.2: the beneficial effect of FMCC on children’s negative affect was compromised only for non-college-educated mothers. For less-educated mothers, sustained financial stress and relatively low social status can increase their negative parenting behaviors (Conger and Donnellan, 2007). These mothers may adopt strict, authoritarian, and other inappropriate styles during mother–child communication; they may also show less support for their children and make more negative comments during the communication process (Baumrind, 1994). Such communication styles tend to lead to more negative affect in children, thus weakening the positive influence of FMCC on children’s affect and obstructing children’s socioemotional development (Gong et al., 2006; Conger and Donnellan, 2007). Conversely, the significant difference between college-educated and non-college-educated mothers supported Hypothesis 4. Numerous studies have demonstrated a significant positive correlation between maternal education and mothers’ sensitivity to their children (Huston and Rosenkrantz Aronson, 2005). In other words, the higher a mother’s level of education, the better she can identify her children’s needs. Consequently, the frequency and method with which she communicates with her children are more likely to meet her children’s emotional needs. More-educated mothers are better able to adopt appropriate methods of communicating with their children in a timely manner, particularly when negative events occur. These mothers help their children better address these negative events and learn to manage their own emotions, effectively reducing negative affect (Fivush, 2007). In addition, college-educated mothers are more likely to express support for their children during mother–child communication, and maternal support is an important protective factor for reducing child depression (Eisman et al., 2015) and anxiety (Brumariu and Kerns, 2015). Furthermore, for college-educated mothers, the positive predictive effect of FMCC on children’s positive affect was not significant, possibly because these mothers have access to a variety of resources because of their high levels of education. Their children generally have a high level of positive affect (Bradley and Corwyn, 2002), which may to some extent obscure the positive effect of FMCC. Although the path coefficient of the college-educated mothers did not reach the level of significance, no significant difference was observed between this path and the path of the non-college-educated mothers. In other words, among the college-educated mothers, FMCC also had a positive predictive effect on children’s positive affect; however, because of the relatively small number of subjects, its predictive effect did not reach a significant level.

Third, for non-college-educated mothers, maternal work hours on workdays significantly directly and positively predicted children’s negative affect; however, no such effect was observed for the college-educated mothers. This result indicated that for non-college-educated mothers, there is in fact some negative effect of maternal work hours on children’s affect, but not through FMCC. A reason may be the types of work that the mothers performed and the emotions that their work evoked. Studies have demonstrated that non-college-educated mothers are typically engaged in low-level jobs with less time flexibility (Galinsky et al., 2011), jobs that are tedious and boring and that often involve heavy manual labor (Hofferth et al., 2000). This type of job is negatively associated with the psychological well-being of employees (Jonge et al., 2001). Thus, it is assumed that spending periods engaged in such jobs increases individuals’ psychological stress and decreases their psychological well-being. Mothers may bring these negative work experiences into their homes and increase their children’s negative affect as a result (Bumpus et al., 2006). By contrast, not only do highly educated mothers have more rewarding jobs but the jobs are also typically more challenging, enabling these women to experience a greater sense of accomplishment and less psychological distress (Chase-Lansdale and Pittman, 2002). Having a positive work attitude makes a mother a good role model for her children, prompts children to cope better with challenges in life and education, and improves their emotional management capability (London et al., 2004), all of which contribute to reducing children’s negative affect. In any case, this is an unexpected and interesting finding, and future studies should explore the underlying mechanism of this relation.

In addition, beyond these differences, our results indicated that for all working mothers, FMCC positively predicted children’s positive affect. (Although the effect for college-educated mothers did not reach the level of significance, there was no significant difference between this effect and the effect for non-college-educated mothers.). This result is consistent with Hypothesis 2.1: in the context of Chinese culture, FMCC benefits children’s positive affect. This result is consistent with many previous studies: FMCC can effectively promote children’s mental health and well-being (Wang et al., 2002; Guo et al., 2014). There may be three underlying reasons for these findings: (1) Mother–child communication facilitates mutual understanding between children and mothers, promotes a sense of closeness and intimacy, and helps establish a positive parent–child relationship (Finkenauer et al., 2002). A positive parent-child relationship not only makes children happier (Davidson and Cardemil, 2009) but also helps them develop positive interpersonal relationships outside the family and promotes their socioemotional development (Pomerantz et al., 2012). (2) Mother–child communication is an important means by which mothers express their concern and support for their children. Evidence from Western countries suggests that such communication helps children feel their mothers’ love and attention, which promotes the development of children’s emotional function (Pomerantz et al., 2007). Chinese mothers are less likely to show their love and support by expressing affection explicitly (Wu et al., 2002); instead, they do so by their efforts to control and govern their children (Chao, 1994). Chinese parental control was determined to positively relate to children’s psychological well-being when parental psychological control was used occasionally (Shek, 2007). Thus, despite differences in cultural norms, Chinese children often perceive support from communication with their mothers, which is beneficial to their psychological well-being. (3) Chinese mothers dedicate more time and attention to their children’s educational activities than do mothers in Western countries (Ng et al., 2007). By mother–child communication, Chinese mothers more frequently communicate the importance of education to their children, stimulate their internal motivation to learn, help them better understand their learning ability and ease their adaptation to school life so that they experience more happiness (Bean et al., 2006; Pomerantz et al., 2012).

Finally, it is noteworthy that maternal work hours on non-workdays negatively predicted FMCC for all working mothers. Although the path coefficient of the college-educated mothers was not significant (*β* = -0.110, *p* = 0.121), there was no significant difference between the two groups. The results indirectly indicated that compared with the negative effect of work hours on workdays, the negative effect of maternal work hours on weekends or holidays on FMCC was more difficult to compensate for. Typically, children have more time at home on weekends and official holidays, which provides greater opportunities for mothers to communicate with their children. Therefore, mothers can use these opportunities to compensate by reducing the time spent on other activities (e.g., leisure, housework) (Bianchi et al., 2006) to decrease the negative effect of their work hours on workdays. However, mothers who must work on weekends and official holidays miss these opportunities, and even if they wanted to use workdays to compensate for the lost time communicating with their children, they would have limited chances to do so because primary school students spend the majority of their time Mondays through Fridays in school (Kim et al., 2016). Taht and Mills (2012) interviewed parents in different careers, and their findings demonstrated that most parents worked on weekends. Parents indicated that overtime work hours on weekends significantly affected the time they spent with their children and reduced their chances to be with, educate, and understand their children, and such losses were difficult to compensate for with shift breaks on weekdays.

## Limitations

The limitations of this study are as follows:

First, the study used a cross-sectional study design, which does not allow for causal inferences. Future research should adopt a longitudinal research design to better reveal the causal relation mechanism between maternal work hours, FMCC, and children’s affect in addition to the developmental dynamics of their relations.

Second, regarding maternal work hours, this study only distinguished between work hours on workdays and non-workdays; it failed to specifically analyze the relation between maternal work hours during different periods (such as daytime, nighttime, early morning, weekends, and holidays) and children’s affect. Future research should examine the duration of maternal work hours during different periods in more detail to further reveal the mechanism influencing the relations between different types of work hours and children’s affect.

Third, although the quality of mother-child communication also plays an important role in child development (Davalos et al., 2005; Kim et al., 2016), this study focused only on its frequency. Therefore, future research should consider both the frequency and the quality of mother-child communication.

Finally, this study only focused on children’s communication with mothers. However, some researchers suggested that maternal care time alone did not predict children’s outcomes; other forms of care time, such as paternal time (Milkie et al., 2015) and daycare center time (NICHD Early Child Care Research Network, 2006; Bulgarelli and Molina, 2016) should be considered. Thus, future studies should further explore the effect of children’s communication with other caregivers.

## Implications for Family Education Practice

First, in addition to intellectual assets, the emotional assets that children acquire during interactions with their parents are also important for their development; emotional assets may help children remain positive when they encounter challenges (Pomerantz et al., 2012). Our results indicated that mother–child communication may be a good way to convey emotional assets to children. Moreover, children in Chinese schools are often under great pressure to perform optimally in their academic work (McCall et al., 2000; Phillipson and Phillipson, 2007); thus, it is necessary for Chinese mothers to communicate with their children more frequently and take children’s affect more seriously.

Second, our results suggested that the frequency of mother–child communication is restricted by increasing maternal work hours, particularly on non-workdays. Therefore, it is beneficial to children if mothers reserve sufficient time for them when they are at home (e.g., weekends, holidays) and participate in activities that facilitate high levels of active engagement and verbal exchange (e.g., sports, arts and crafts).

Finally, our results indicated that for non-college-educated mothers, more mother–child communication was not associated with less negative affect. This result reminds us to pay more attention to the families with less educated mothers, examining why the protective effect of mother–child communication on children’s negative affect disappears and help them to better implement the beneficial functionality of mother–child communication.

## Conclusion

This study contributes to the literature by offering direct evidence of the complex relation between maternal work hours, FMCC and children’s affect for two different groups of mothers: college-educated mothers and non-college-educated mothers. First, increasing work hours theoretically limits mothers’ time with their children (Hoover-Dempsey and Sandler, 2005). In general, our results support this assumption, particularly for the work hours on non-workdays. However, there is an exception: for college-educated mothers, the work hours on workdays cannot negatively predict FMCC. This exception indicates that college-educated mothers may attempt to compensate for the loss of time for parent–child activities because of their work hours, but only on workdays. Second, our results are mostly consistent with previous studies: that mother-child communication is beneficial to children’s affect (Kerr et al., 1999; Bean et al., 2006). However, there is also an exception: for non-college-educated mothers, the negative effect of FMCC on children’s negative affect disappeared. This result indicates that non-college-educated mothers may not successfully take advantage of their communication with children to reduce children’s negative affect. Finally, we observed that non-college-educated mothers’ work hours on workdays directly and positively predict children’s negative affect. Because this effect was not because of FMCC, there may be other factors that contribute to this relation that were not included in this study.

## Author Contributions

HZ: designing and drafting the work; acquisition, analysis, and interpretation of data for the work. BL: acquisition, analysis of data; revising manuscript. XG and CL: acquisition, analysis of data. BQ: acquisition of data. WH: revising manuscript. ZL and LL: substantial contributions to design of the work; final approval of the version to be published.

## Conflict of Interest Statement

The authors declare that the research was conducted in the absence of any commercial or financial relationships that could be construed as a potential conflict of interest.
